# Cryptanalysis of an Image Encryption Algorithm Based on Random Walk and Hyperchaotic Systems

**DOI:** 10.3390/e24010040

**Published:** 2021-12-25

**Authors:** Haiju Fan, Heng Lu, Chenjiu Zhang, Ming Li, Yanfang Liu

**Affiliations:** 1 College of Computer and Information Engineering, Henan Normal University, Xinxiang 453007, China; 121064@htu.edu.cn (H.F.); zcjmail2021@163.com (C.Z.); liming@htu.edu.cn (M.L.); 121101@htu.edu.cn (Y.L.); 2 Engineering Lab of Intelligence Business & Internet of Things, Xinxiang 453007, China; 3 Big Data Engineering Lab of Teaching Resources & Assessment of Education Quality, Xinxiang 453007, China; 4 Key Laboratory of Artificial Intelligence and Personalized Learning in Education of Henan Province, Xinxiang 453007, China

**Keywords:** cryptanalysis, random walk, hyperchaotic systems, scrambling-diffusion

## Abstract

Recently, an image encryption algorithm based on random walk and hyperchaotic systems has been proposed. The main idea of the original paper is to scramble the plain image by means of random walk matrix and then to append diffusion. In this paper, the encryption method with security holes is analyzed by chosen plaintext attack. In addition, this paper improves the original encryption algorithm. The experimental and simulation results show that the improved algorithm has the advantages of the original and can improve the ability to resist attack.

## 1. Introduction

With the development of the Internet and cryptography, communication encryption has attracted the attention of many researchers and has been applied in many fields [1,2]. At the same time, the number of images transmitted over the Internet is increasing all the time [3]. However, they spread through such insecure channels and are easy to be intercepted by some technical means, which leads to the disclosure of many people’s privacy and security problems such as the random modification of digital image information [4]. In recent years, researchers have proposed many image encryption algorithms, especially those based on chaos, mainly because chaos dynamics have various advantages applicable to cryptography, such as pseudo-randomness, ergodic property, sensitivity to initial values, and controlling parameters. When Fridrich [5] first applied the structure of permissive diffused junction encryption to image encryption, many chaotic image encryption schemes based on it appeared [6,7,8,9,10]; however because the encryption algorithms have security holes, or only use one round encryption, it is extremely vulnerable to be cracked by chosen-plaintext attacks [11,12,13].

Fridrich’s encryption algorithm was broken by Solak [14], using chosen ciphertext attacks. Hua et al. [15] proposed a multi-round encryption algorithm, and Yu et al. [16] solved the linear relationship of the encryption algorithm. No matter how many rounds of encryption are carried out, security loopholes can always be found without being related to plaintext. Li et al. [17] improved the security of the system through multiple rounds of encryption related to plaintext. In addition, many researchers [18,19,20,21,22] sacrifice the efficiency of encryption to obtain encrypted security groups. However, some plaintext-related encryption algorithms [23,24,25] are attacked by chosen plaintext because of the small key space. Therefore, some researchers improved the traditional scramble diffusion structure by combining simultaneous scramble diffusion and cross diffusion [26,27,28] to increase the difficulty of an attack. Nowadays, researchers [29,30,31,32] integrate the plaintext-related hash function into the encryption algorithm to increase the key space, resulting in the failure of traditional chosen plaintext attacks. The traditional encryption method of the scrambling-diffusion structure becomes more and more complicated.

An image encryption scheme based on a random walk and two hyperchaotic systems is proposed. On the basis of a bit-level encryption scheme, the author has designed a more complex encryption algorithm with randomness and unpredictability. The encryption scheme can be simplified into three stages: the key generation stage, the scrambled stage, and the diffusion stage. In the key generation stage, two pseudo-random sequences are first generated, and are then used to scramble the pixel position in the scrambled stage and the diffusion method to change the pixel value, respectively. Due to the random walk matrix used for the scramble, the initial position and direction of the motion are controlled by the random numbers obtained from the chaotic system, with a significantly better safety performance than other encryption schemes mentioned above.

Our paper analyzes the security of the encryption system, and, through the corresponding theoretical analysis and experimental verification, we find that there are some security loopholes in the original scheme. In this paper, the cryptanalysis of the scheme is carried out, and the attack method is proposed to successfully break the encryption scheme of the original paper. The theoretical analysis and simulation results verify the high efficiency and feasibility of the proposed attack scheme. The special point of this paper is to verify whether the chaotic encryption of multiple random sequences in the scrambled stage and diffusion stage is invalid. The method in this paper can be used to attack and obtain the original image easily. Many papers have used more complex chaotic system generators and more chaotic sequences, but this does not change the security of encryption significantly. We think that the encryption algorithm should be changed to combine the chaotic sequence with the image more closely. Beyond that, the rest of our paper is as follows: The Section 2 outlines the original encryption scheme. The Section 3 analyzes its security vulnerabilities and makes preparations. In the Section 4, the chosen plaintext attack scheme is used for simulation and experiment. In the Section 5, the algorithm is improved. The Section 6 analyzes the security of the improved algorithm.

## 2. Review of the Original Image Encryption Scheme

**Figure 1:**
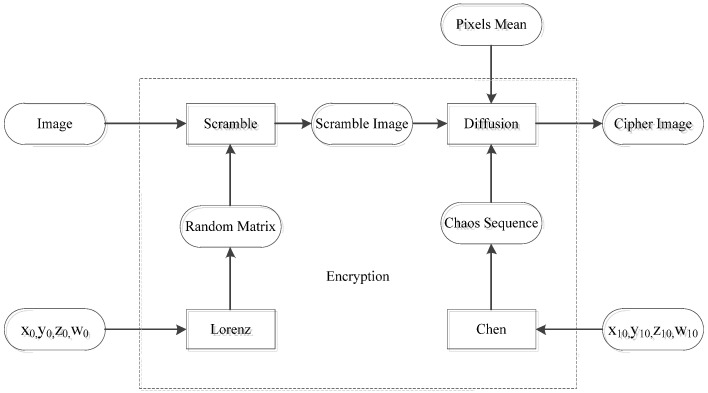
Original encryption scheme.

The flow chart of the original scheme is shown in Figure 1. In the scheme, the image is first divided into blocks, and is then scramble and diffused. In the scrambled stage, the random walk matrix is generated by a Lorenz chaotic system. The blocks are first scramble internally and then scrambled between blocks, so as to update pixel positions. In the diffusion stage, the chaotic system of the Chen type is used to generate the sequence, and the average pixel is used as the initial diffusion value to encrypt the scrambled image.

### 2.1. The Secret Key Generation Stage

A chaotic system is used to obtain the key sequences. The hyperchaotic Lorenz system and Chen’s hyperchaotic system are represented by Equations (1) and (2):

(1) $$\{\begin{array}{c} x=\alpha \left(y-z\right)+w,\\ y=\gamma x-y-xz,\\ z=xy-\beta z,\\ w=-yz+\eta w. \end{array}$$

where *α* = 10, *β* = 8/3, *γ* = 28 and *η* = 1 presents a chaotic state.

(2) $$\{\begin{array}{c} x=a\left(y-x\right),\\ y=-xy+nx+my-w,\\ z=xy-bz,\\ w=x+k. \end{array}$$

where *a* = 36, *b* = 3, *m* = 28, *n* = 16, and *k* = 0.2 presents a chaotic state.

Note that the variable and constant symbols used in this section represent only the standard generation formula for chaotic systems and do not have the same meaning as the symbols used in the full text.

### 2.2. Random Walk Matrix

Step 1: Initial keys *x*_0_, *y*_0_, *z*_0_, *w*_0_, by iterating over a hyperchaotic Lorenz system *N*_0_ + 8 ∗ *M* ∗ *N* time, to generate several pseudo-random sequences *X*, *Y*, *Z* and *W*. *M* and *N* are the size of the encrypted image, and *N*_0_ is obtained from Equation (3).

(3) $$N_{0}=\mathrm{mod}\left(h,1500\right)$$

where *h* is a decimal hash value.

Step 2: The initial position of the random walk (*Px*, *Py*) is determined by the random sequences *Y* and *Z*:

(4) $$\{\begin{array}{c} Px\left(i\right)=\mathrm{mod}\left(floor\left(y\left(i\right)\times 10^{13}\right),m\right)+1,\\ Py\left(i\right)=\mathrm{mod}\left(floor\left(z\left(i\right)\times 10^{13}\right),n\right)+1. \end{array}$$

Step 3: The direction of the random walk is controlled by *X*:

(5) $$X\left(i\right)=\mathrm{mod}\left(floor\left(x\left(i\right)\times 10^{13}\right),8\right)$$

Step 4: According to the initial position and direction, *M* ∗ *N*/(*m* ∗ *n*) random walk matrices are obtained.

### 2.3. Scrambling

Scrambling inside blocks: In this stage, the pixel position in each sub-block image is scramble based on the random walk matrix.

For *i* = 1, 2, …, (*M* ∗ *N*)/(*m* ∗ *n*) and *j* = 1, 2, …, *m* ∗ *n*.

(6) $${B^{\prime }}_{i}\left(j\right)=B_{i}\left(L\left(j\right)\right)$$

where **B** is the sub-block and **L** is the sequence obtained by sorting the random walk matrix **A**.

Scrambling between blocks: After the scramble of the previous step, a scramble between blocks is adopted; in other words, the pixels of the block are scramble with those of the other blocks.

For **I** = 1, 2, …, (*M* ∗ *N*)/(*m* ∗ *n*).

(7) $$k1\left(i\right)=\mathrm{mod}\left(W\left(i\right),\frac{M\times N}{m\times n}-i\right)+1$$

(8) $$D={B^{\prime }}_{i}\left(k2\left(i\right)\right).$$

where, *k*2 is the sequence obtained after sorting *k*1, and *D* is the scrambled image.

### 2.4. Diffusion

The original keys are *x*_10_, *y*_10_, *z*_10_, *w*_10_. By iterating the hyperchaotic Chen system *N*_0_ + 8 ∗ *M* ∗ *N* times, four pseudo-random sequences *x*_1_, *y*_1_, *z*_1_, and *w*_1_ are generated, which are merged into two chaotic sequences *X* and *Z*.

(9) $$\{\begin{array}{c} X_{1}\left(i\right)=\mathrm{mod}\left(floor\left(x_{1}\left(i\right)+y_{1}\left(i\right)\times 10^{14}\right),256\right),\\ Z_{1}\left(i\right)=\mathrm{mod}\left(floor\left(z_{1}\left(i\right)+w_{1}\left(i\right)\times 10^{14}\right),256\right). \end{array}\#9$$

Use *X*, *Z* chaotic sequences for diffusion:

(10) $$\{\begin{array}{c} C\left(1\right)=\mathrm{mod}((X_{1}\left(1\right)+Z_{1}\left(1\right)),256)\\ ⊕\mathrm{mod}\left(\left(D\left(1\right)+aver\right),256\right),\\ C\left(i\right)=\mathrm{mod}((X_{1}\left(i\right)+Z_{1}\left(i\right)),256)\\ ⊕\mathrm{mod}\left(\left(D\left(i\right)+C\left(i-1\right)\right),256\right). \end{array}$$

where **D** is the scrambled image and *aver* = *Sum*(*D*)/(*M* ∗ *N*).

## 3. Cryptanalysis

**Figure 2:**
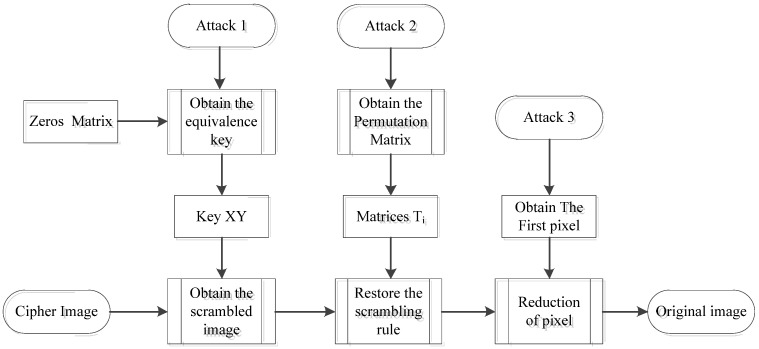
The process of attack.

As is known, Kehoff’s principle of the security of a cryptographic system depends entirely on the key, rather than the complexity of the cryptographic system itself. That is to say, the attacker would know all the information in the original encryption scheme except the secret key. Considering that the cryptographic system proposed by Xu et al. uses a pseudo-random sequence to scramble the image and perform the XOR operation, and although the average value of the image is used as the initial parameter of diffusion, it only carries out one forward diffusion, thus it can be divided into two steps to obtain the plaintext image. The following Figure 2 shows the attack flow.

### 3.1. Obtain Diffusion Key

**Figure 3:**
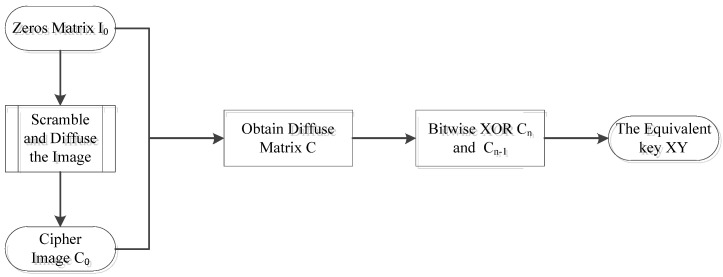
Flowchart of obtaining the key.

In this section, we discuss how to obtain the keys needed for diffusion. The key generation in the original text has good key space and sensitivity, and because the average value of the image is used as the initial diffusion and the first bit diffusion result is applied to the subsequent diffusion sequence, the diffusion of each image is not the same, and it cannot be obtained simply through the full zero or constant image. However, because of the correlation between the before and after pixel diffusion, we can analyze the diffusion key through the cipher image. Figure 3 is the flow chart showing how to obtain the key.

In the figure above, we describe the process of obtaining the equivalent diffusion key *XY* through a flowchart. We found that, when the attacker chose a full zero image such as the plaintext image, since all the pixels in **I**_0_ are zero, the plaintext image is not affected by the scrambled process. Therefore, image **I**_0_ will be chosen to obtain the corresponding scrambled image **D**_0_, which is also going to be a full zero image. Then, the diffusion is used to obtain the ciphertext image **C**_0_; thus, the corresponding ciphertext image sequence is obtained, which is the equivalent diffusion key *XY*. In this way, the scrambled image of any chosen plaintext image can be obtained by diffusion key *XY*.

The process of obtaining the key is to first construct an image **I**_0_ of full zero pixels, and to obtain the corresponding ciphertext image **C**_0_. If you use a full zero image to encrypt, that means you can skip the scrambled phase and reach the corresponding scrambled matrix **D**_0_. The encryption process uses the addition of two chaotic sequences, *X* and *Z*, which can be regarded as a chaotic sequence *XY*. Therefore, according to the diffusion rule, the key can be obtained by Equation (11).

(11) $$\{\begin{array}{c} XY\left(1\right)=C_{0}\left(1\right)⊕(D_{0}\left(1\right)+aver),\\ XY\left(i\right)=C_{0}\left(i\right)⊕(D_{0}\left(i\right)+C_{0}\left(i-1\right)). \end{array}$$

where, **C**_0_ is the cipher image corresponding to full zero image **D**_0_ and the average *aver* of full zero image is also zero.

Thus, it can be further deduced:

(12) $$\{\begin{array}{c} XY\left(1\right)=C_{0}\left(1\right),\\ XY\left(i\right)=C_{0}\left(i\right)⊕C_{0}\left(i-1\right). \end{array}$$

After revealing the diffusion key *XY*, through the final ciphertext image **C**, the attacker only needs to simply perform the XOR and the inverse addition and subtraction operation to obtain the plaintext image. Algorithm 1 describes in detail the process of obtaining the key *XY*, and Algorithm 2 is the process of obtaining only scrambled image **D**.
**Algorithm 1.** Obtain secret key *XY*.Input: full zero image **I**_0_ Output: The key *XY*1:procedure key(**I**_0_)2: *M*, *N* ← size(**I**_0_)3:**C**_0_ ← encry(**I**_0_)4:*XY*(1) ← **C**_0_(1)5: for *i* from 2 to *M* ∗ *N*6:    *XY*(*i*) ← *bitxor*(*C_i_*,*C*_*i*−1_)7:end8:end procedure

**Algorithm 2.** Obtain Scrambled images **D**.Input: key *XY*, cipher image **C** Output: Scrambled images **D**1:procedure *Scram*(*XY*,**C**)2:*M*, *N* ← *size*(**C**)3: for *i* from 2 to *M* ∗ *N*4:   *D*’(*i*) ← *C*(*i*)⊕*XY*(*i*)5:   if *D*’(*i*) > *C*(*i* − 1)6:     *D*(*i*) = *D*’(*i*) − *C*(*i* − 1)7:   else8:     *D*(*i*) = *D*’(*i*) + 256 − *C*(*i* − 1)9:   end10: end11:end procedure

### 3.2. Gets the Scrambling Rule

**Figure 4:**
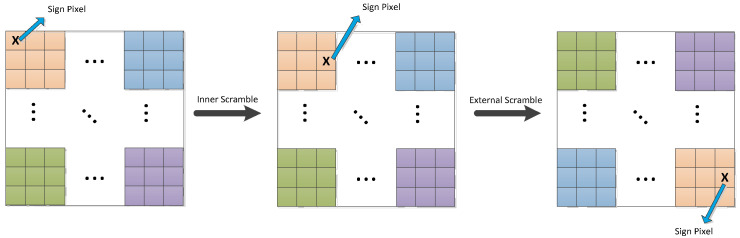
The affection of two-round scrambling.

It can be seen from the encryption process in Section 2 of this paper that the input pixel is scramble twice in the scrambled process; that is, the pixel blocks are scramble inside and between the blocks. However, in a mathematical sense, two-round scramble have exactly the same encryption effect as one-round scramble, as shown in Figure 4. In other words, no matter how complicated the scramble process is, it simply changes the order of the pixels. For the ordinary image encrypted by this scheme, only two construction matrices can be used to obtain the scrambled rules. Color images or greater images only need to add an extra choice matrix in this changed extension.

In this section below, our main goal is to attack the scrambled phase of the original encryption scheme. In this step, as long as the scrambled image **D** is restored to the normal image **I** before scramble, the original password system is successfully attacked. It is important to note that any existing scrambled algorithm only changes the position of the pixel, not the value of the pixel, which allows an attacker to test the image to obtain a scrambled rule. Therefore, the key point is that, as long as we can find the original position and the new position of each pixel of the original image after the scrambled stage, we can obtain the equivalent scrambled matrix **H**, thus successfully breaking the scrambled stage of the original cipher system.

We chose a 256 × 256 plaintext image and can use two pairs of specially selected plaintext images to obtain the value of n, as follows:

(13) $$n\geq \log_{256}256\times 256$$

Considering that the original cryptographic system uses two scrambling, the final scramble can be regarded as the corresponding position obtained by one scramble. We define two marker matrices to mark the position of each pixel of the ordinary image by Equations (1) and (2):

(14) $$m_{1}=\left[\begin{array}{ccccc} 0 & 0 & 0 & \cdots & 0\\ 1 & 1 & 1 & \cdots & 1\\ \vdots & \vdots & \vdots & \vdots & \vdots \\ 255 & 255 & 255 & \cdots & 255 \end{array}\right]$$

(15) $$m_{2}=\left[\begin{array}{ccccc} 0 & 1 & 2 & \cdots & 255\\ 0 & 1 & 2 & \cdots & 255\\ \vdots & \vdots & \vdots & \vdots & \vdots \\ 0 & 1 & 2 & \cdots & 255 \end{array}\right]$$

The size of **m**_1_ and **m**_2_ is 256 × 256. First, the two marker matrices are encrypted to obtain the corresponding encryption graphs **C**_m1_ and **C**_m2_. However, when the corresponding scrambled map is obtained through the diffusion secret key *XY*, it is necessary to take into account the average *aver* of **C**_m1_ and **C**_m2_ after the encryption of **m**_1_ and **m**_2_, which is replaced by *f*1 and *f*2, respectively. It can be obtained through the following formula:

(16) $$\{\begin{array}{c} f_{1}=round\left(\overset{M}{\underset{i=1}{\sum }}\overset{N}{\underset{j=1}{\sum }}m_{1}\right)/M\times N,\\ f_{2}=round\left(\overset{M}{\underset{i=1}{\sum }}\overset{N}{\underset{j=1}{\sum }}m_{2}\right)/M\times N. \end{array}$$

When the constructed matrices **m**_1_ and **m**_2_ are used to obtain the scrambled graph through diffusion matrix, the following formula is needed to obtain the first pixel bit of **O**_m1_ and **O**_m2_ of the scrambled graph:

(17) $$\{\begin{array}{c} O_{m1}\left(1\right)=(C_{m1}\left(1\right)⊕XY\left(1\right))-f_{1},\\ O_{m2}\left(1\right)=(C_{m2}\left(1\right)⊕XY\left(1\right))-f_{2}. \end{array}$$

Next, Algorithm 3 is used to encrypt image **C**_m1_ and **C**_m2_ to obtain scrambled matrix **O**_m1_, except the first pixel, and all remaining pixels of **O**_m2_. After obtaining the scrambled graphs of the two marker matrices, **m**_1_ and **m**_2_, we can obtain the relative scrambled position of the ciphertext image **C** by an equivalent scrambled matrix **H**. However, first, the pixel ranges of two scrambled images **O**_m1_ and **O**_m2_ are converted into a matrix of 65,536 possible pixel positions. The following formula can be used:

(18) $$H=256\times O_{m1}+O_{m2}$$

Thus, we can obtain the scrambled position of each pixel from 1 to 65,536; that is, shrink it from any possible pixel position to correspond to the original pixel one by one, so as to accurately determine the unique scrambled position of the original ordinary image. The specific algorithm is Algorithm 3.
**Algorithm 3.** Obtain the permutation rule **H**.Input: Select the image **m**_1_,**m**_2_, key *XY* Output: permutation rule **H**1:procedure *Rule*(**m**_1_, **m**_2_)2: *M*, *N* ← *size*(**C**)3: **C**_m1_, **C**_m2_ ← *encry*(**m**_1_, **m**_2_)4: **O**_m1_, **O**_m2_ ← *Algorithm* 2(**C**_m1_, **C**_m2_)    //Obtain Scrambled images5: for *i* from 1 to *M*6:   for *j* from 1 to *N*7:     H(*i*,*j*) ← 256 ∗ **O**_m1_ (*i*,*j*) + **O**_m2_ (*i*,*j*)8:   end9: end10:end procedure

### 3.3. Restore Plaintext Image

Because the original plaintext image **I** is diffused when the first pixel diffuses, *aver* is used to obtain encryption **C**. Unlike the *aver* used for the original plaintext image **I**, this results in the diffuse value being an unknown pixel value. But subsequent diffusion is an *aver* independent process, so we can temporarily set the first pixel value of the original image to the unknown number *x*_first_. After the diffusion reduction of *XY* chaotic sequence, only scrambled matrix **D** is obtained, and then the equivalent scrambled matrix **H** is used. **D** is restored to the image **I**_p_ except the first pixel value. Finally, *x*_first_ is solved and restored to the corresponding position of the original image. In other words, plaintext image can be restored.

**Figure 5:**
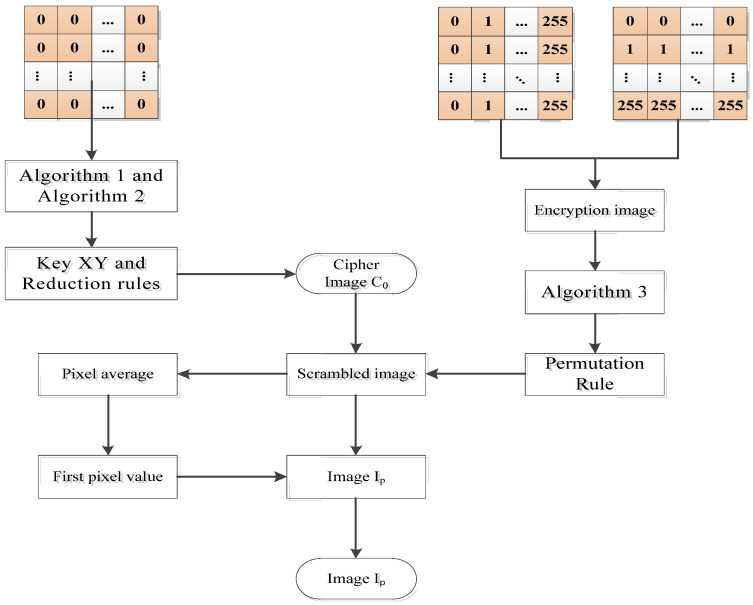
The process of restoring a plaintext image.

Since the diffusion sequence *XY* and the scrambled rule have been obtained, it is easy to obtain the plaintext image **I**_p_ except the first one. The specific recovery process is shown in Figure 5, which will not be described in detail here. The following is the specific process of finding the first pixel *x*_first_.

The *aver* of the original plaintext image **I** is as shown in the following formula:

(19) $$aver=\overset{M}{\underset{i=1}{\sum }}\overset{N}{\underset{j=1}{\sum }}\frac{I\left(i,j\right)}{M\times N}$$

When the original encryption system encrypts, the cipher image encrypted by plaintext **I** is **C**, which can be transformed into the following equation:

(20) C(1)=XY(1)⊕(D(1)+aver)

At this point, *C*(1) in the original cipher image **C** is known. After the previous steps, the chaotic sequence can be obtained as *XY*. Let us assume the first pixel value *x*_first_ and convert it into:

(21) $$x_{first}=\left(C\left(1\right)⊕XY\left(i\right)\right)-aver$$

According to the previous steps, we have obtained all pixel values except *x*_first_, then we can know that *aver* is also equal to:

(22) $$aver=\left[x_{first}-\overset{M}{\underset{i=1}{\sum }}\overset{N}{\underset{j=2}{\sum }}I\left(i,j\right)\right]/\left(M\times N\right)$$

From Equations (21) and (22), we can obtain two formulas to get the value of *x*_first_ and combine them, as shown in Equation (23):

(23) $$\begin{array}{c} x_{first}=round\{[M\times N\times \left(D\left(1\right)+256\right)\\ -\overset{M}{\underset{i=1}{\sum }}\overset{N}{\underset{j=1}{\sum }}I_{p}-D\left(1\right)]/\left(M\times N+1\right)\} \end{array}$$

**Figure 6:**
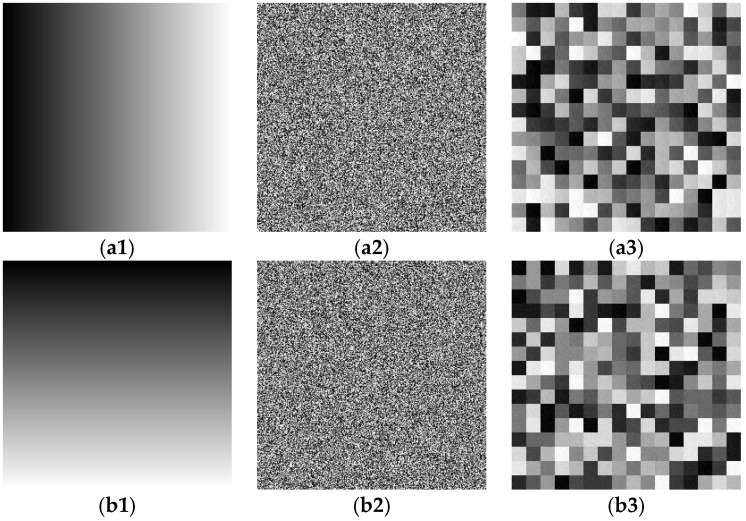
Chosen plaintext attack to obtain the scrambled rule: (**a1**) and (**b1**) is the chosen plaintext image; (**a2**) and (**b2**) is the corresponding cipher image; (**a3**) and (**b3**) is the retrieved only scrambled image corresponding to the second column.

**Figure 7:**
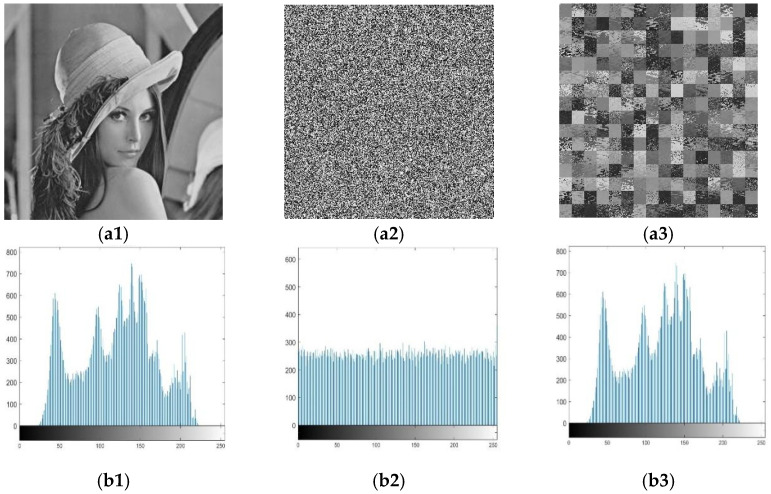
(**a1**) is a Lena image; (**a2**) is cipher image corresponding to (**a1**); (**a3**) is only scrambled image obtain from (**a3**); (**b1**) is histogram corresponding to (**a1**); (**b2**) is histogram corresponding to (**a2**); (**b3**) is histogram corresponding to (**a3**).

Figure 6 shows the result of the chosen plaintext attack to obtain only scrambled images. In Figure 6, the first column is the plaintext image, the second column is the final ciphertext image, and the third column is the resulting only scrambled image. Figure 6(a1,b1) shows two different selected plaintext images; Figure 6(a2,b2) is the final ciphertext image of the plaintext image in Figure 6(a1,b1) after the original encryption scheme. Figure 6(a3,b3) is the only scrambled image obtained after decrypting the final ciphertext image Figure 6(a2,b2) using the diffusion key XY shown above. As long as a unique scrambled image is obtained, the original position can be restored by mapping, and the scrambled rules can be obtained to find the original plaintext image, as described above. In addition, in this section, we use an element that marks a particular pixel position (1,1) of the original plaintext image to restore the x value. Figure 7 shows the histogram display of the scrambled image of the original image. From the perspective of the result, the pixel value is consistent with that of the original image, which proves that the scrambled image of the original image is successfully obtained.

### 3.4. Summary

The detailed process is shown in the following steps:

Step 1: Determine the number of chosen plaintext images by Equation (12) and construct the equivalent key *XY* by selecting the plaintext image, as shown in Figure 3, so as to obtain the scrambled image **D** of the original cipher image **C**.

Step 2: Next, obtain the scrambled images **O**_m1_ and **O**_m2_ corresponding to the chosen plaintext **m**_1_ and **m**_2_ by the equivalent key *XY*. Algorithm 3 is used to obtain the equivalent scrambled matrix **H** corresponding to the original scrambled stage. After recording the scrambled operation, select the new pixel position of each line of the plaintext image **m**_1_ and **m**_2_.

Step 3: By scrambling the matrix **H** equivalently, obtain the plaintext image **C** of the only scrambled image **D**.

Step 4: Finally, the plaintext image **I** corresponding to the cipher image **C** is obtained by calculating the value of the pixel in the original position scramble by the first pixel of the image **I**_p_.

**Figure 8:**
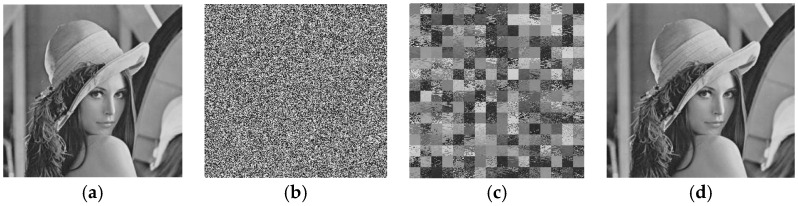
Effectiveness of the attacks: (**a**) The original Lena image; (**b**) The cipher image corresponding to (**a**); (**c**) The scrambled images retrieved from (**b**); (**d**) The final recovered image.

In addition, the simulation experiments were carried on a personal computer equipped with an Intel Core i5-10210U 2.11GHz CPU and 16 GB memory capacity, which verifies the effectiveness and feasibility of the proposed attack scheme. Matlab software R2020a was used in the simulation experiments. In order to prove the feasibility and correctness of our scheme, we used the same initial and control parameter values to carry out the simulation experiments. The proposed algorithm can successfully attack all kinds of grayscale images encrypted by the original cryptographic system, and the average usage time is 12.4573 s. We use the size of 256 × 256 ordinary images for simulation experiments, including the size of 256 × 256 “Lena” image, the size of 256 × 256 “Airfield” image, the size of 256 × 256 “Dollar” image, and the size of 256 × 256 “Pepper” image. All test images were from the USC-SIPI image database. The experimental results are shown in Figure 8.

## 4. Improvement

**Figure 9:**
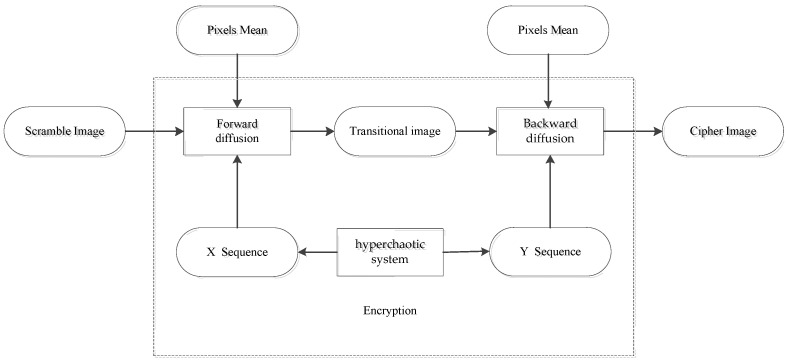
The diffusion process of improved algorithm.

In the original encryption scheme, the diffusion is carried out according to the XOR result of the former bit, but the diffusion result of the former bit is exposed in the cipher image, therefore it is very insecure. In order to hide the relationship between diffused pixels, two-way diffusion can be adopted. Without changing the original scheme secret key and encryption framework, forward diffusion and backward diffusion are carried out through two chaotic sequences *X*_1_(*i*) and *Y*_1_(*i*) to improve security, as shown in Figure 9.

Step 1: Scramble the image, as shown in Section 2.3.

Step 2: The chaotic sequence is obtained according to Equation (2): *X*_1_(*i*), *Y*_1_(*i*).

(24) $$\{\begin{array}{c} X_{1}\left(i\right)=\mathrm{mod}\left(floor\left(x_{1}\left(i\right)+y_{1}\left(i\right)\times 10^{14}\right),256\right),\\ Y_{1}\left(i\right)=\mathrm{mod}\left(floor\left(z_{1}\left(i\right)+w_{1}\left(i\right)\times 10^{14}\right),256\right). \end{array}$$

Step 3: Forward diffusion:

(25) $$\{\begin{array}{c} C\left(1\right)=\mathrm{mod}(X_{1}\left(1\right),256)⊕\mathrm{mod}\left(\left(D\left(1\right)+aver\right),256\right),\\ C\left(i\right)=\mathrm{mod}(X_{1}\left(i\right),256)⊕\mathrm{mod}\left(\left(D\left(i\right)+C\left(i-1\right)\right),256\right). \end{array}$$

where **D** is diffusion result, and **C** is a matrix of forward diffusion. *aver* = *Sum*(*D*)/(*M* ∗ *N*), *i* = 2, 3, 4, …, *M* ∗ *N*.

Step 4: Backward diffusion:

(26) $$\{\begin{array}{c} T\left(p\right)=\mathrm{mod}(Y_{1}\left(p\right),256)⊕\mathrm{mod}\left(\left(C\left(p\right)+aver\right),256\right),\\ T\left(i\right)=\mathrm{mod}(Y_{1}\left(i\right),256)⊕\mathrm{mod}\left(\left(C\left(i\right)+C\left(i+1\right)\right),256\right). \end{array}$$

where **C** is the forward diffusion result, **T** represents the backward diffusion result, *p* = *M* ∗ *N*, *i* = *p* − 1, *p* − 2, *p* − 3, …, 1.

## 5. Performance of the Improved Cryptosystem

In this section, the experimental results are given and the performances of the improved cryptographic system are analyzed. Here, we obtain the normal image from the USC-SIPI image database. In addition, other size images can also be effectively encrypted by the proposed algorithm, which is not tested very much in this paper.

We tested the improved encryption method in the following five parts: Histogram Analysis, Correlation Analysis, Information Entropy Analysis, Differential Attack Analysis, and Computational and Complexity Analysis. Since the chaotic sequence used in this paper and the generated key belong to the original encryption scheme and have undergone a lot of tests, the randomness of the sequence and the key space are not tested here.

### 5.1. Histogram Analysis

Histogram analysis can be very intuitive to observe the distribution of pixels. A good encryption method, its histogram inevitably shows the characteristics of pixel homogenization. Compared with the original scheme, the improved scheme inherits the advantages and improves the security.

**Figure 10:**
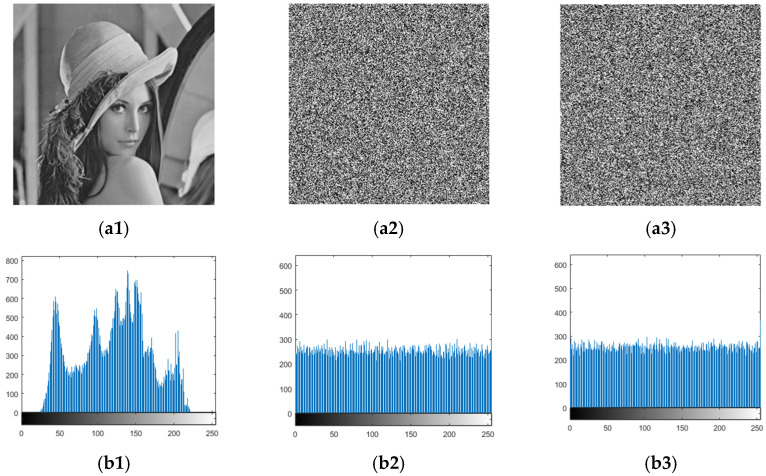
Histogram comparison of two encryption methods: (**a1**) is original image and (**b1**) is its histogram; (**a2**) is original encrypted image and (**b2**) is its histogram; (**a3**) is improved encrypted image and (**b3**) is its histogram.

Figure 10 shows the result of the original encryption scheme and the improved scheme. (a1) Represents a plaintext image and (b1) is the corresponding histogram; (a2) represents the original encryption image and (b2) is the corresponding histogram; (a3) represents the improved encrypted image and (b3) is the corresponding histogram. It can be seen from the histogram that the improved scheme has better chaos. We verified the visual uniformity by calculating the histogram variance. The histogram of the original scheme has a pixel variance of 311.5029, and variance after improvement is 276.2195. Obviously, the improved scheme has a smoother distribution in the histogram.

### 5.2. Correlation Analysis

**Figure 11:**
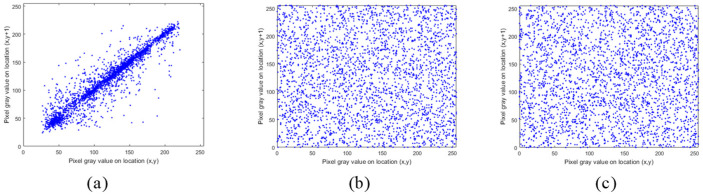
Correlation distribution of adjacent pixels (horizontal direction): (**a**) represents original images; (**b**) represents original encrypted image; (**c**) represents improved encrypted image.

**Figure 12:**
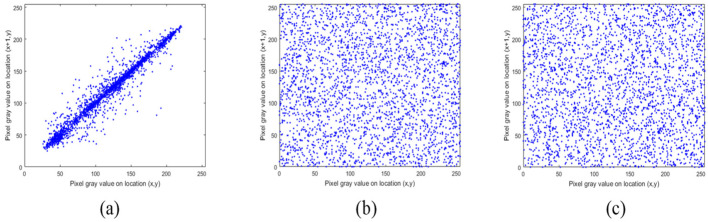
Correlation distribution of adjacent pixels (vertical direction): (**a**) represents original images; (**b**) represents original encrypted image; (**c**) represents improved encrypted image.

**Figure 13:**
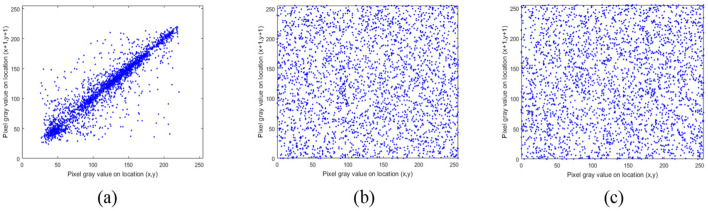
Correlation distribution of adjacent pixels (diagonal direction): (**a**) represents original images; (**b**) represents original encrypted image; (**c**) represents improved encrypted image.

**Figure 14:**
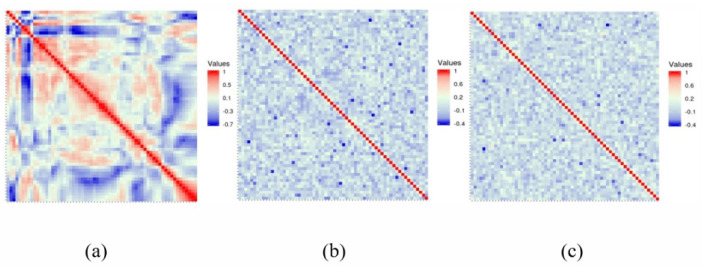
Correlation heat map: (**a**) represents original images; (**b**) represents original encrypted image; (**c**) represents improved encrypted image.

The pixels of ordinary images usually have high neighborhood correlation, which can be weakened by the encryption algorithm. In addition, better algorithms lead to weaker correlations. The correlation between the adjacent pixels of the plaintext image and the ciphertext image is compared, and the results are shown in Figure 11, Figure 12 and Figure 13. The correlation heat map of the images is shown in Figure 14. Relative to the correlation, the improved algorithm is comparable with the original one.

According to the experimental analysis, Table 1 shows the image correlation coefficients obtained by different algorithms. When the correlation coefficient of the ordinary image approaches 1, and the correlation coefficient of the encrypted image approaches 0, then we can consider that it has a good encryption effect. It can be seen from Table 1 that the coefficients of the improved algorithm approach 0 infinitely in horizontal, vertical, and diagonal directions. As can be seen from the above experimental results, the improved algorithm can break the correlation between adjacent pixels through encryption. In Table 1, we select four images for correlation analysis, and the horizontal and vertical coefficients in most images are reduced. Although the diagonal direction of some images did not decrease, the average value is smaller as a whole.

### 5.3. Information Entropy Analysis

In the field of image encryption, information entropy is an important index to reflect the randomness of information. A good secure cipher image has great statistical randomness and information entropy. The standard of information entropy is 8, which means that, when the password image is close to this value, its distribution is approximately random. Therefore, different encryption algorithms will obtain different information entropy; of course, the same algorithm will change due to different images. The experimental results are shown in Table 2. The information entropy of all encryption algorithms is close to 8, and they all pass the randomness test. The average entropy of the four images is 7.9968, which is an acceptable standard error.

### 5.4. Differential Attack Analysis

Resistance to differential attacks is another security criterion, and its ability lies in the sensitivity of ordinary images to change. In other words, if the encrypted algorithm is sensitive to changes in pixel values, it is well resistant to differential attacks. In order to test the ability of the improved algorithm, the following representative images were selected and the pixels in them were randomly selected to obtain the average value. As shown in Table 3, NPCR values were close to the ideal value (99.6094%), and UACI values ranged from 33.3% to 33.6%. It can be seen from the table that there is not much difference in the ability of the two encryption schemes to resist differential attacks. Therefore, changing one pixel in the ordinary image will affect almost all of the pixels in the cipher image, which shows that the scheme has better security against differential attack.

### 5.5. Computational and Complexity Analysis

In Table 4, we conducted experiments on the original encryption algorithm and the improved algorithm and obtained their encryption execution time, and the execution environment is shown in Section 3. In order to improve the accuracy, we encrypt different natural images several times and use the difference methods to obtain the average value for comparison. In addition, when the improved algorithm is executed under different images and environments, the execution time is almost the same as that of the original scheme.

## 6. Conclusions

The encryption scheme based on the random walk and hyperchaotic system has the advantage of the average value of pixels related to plaintext, which can be used as the diffusion basis to resist the general attack scheme. However, our proposed method can effectively break the encryption mechanism. The experimental results verify the effectiveness of cryptanalysis, and the execution speed also meets the requirements. Therefore, in order to improve the security of the encryption method, we give the improved diffusion course. The key space and number are the same as the original scheme, and the execution efficiency of the algorithm is almost the same as that of the original scheme, but it greatly improves the security of the cryptographic system. From the experimental results, the encrypted image of the new algorithm is sufficiently chaotic. However, the encryption method in this paper is not just to prove that it is more chaotic than other methods or has higher performance. Note that the key to this article is hard-to-crack security. Compared with the XOR of the original algorithm, this paper applies two-step diffusion to all pixels from beginning to end, and uses another chaotic sequence to reverse diffusion, which gives the new algorithm an order of magnitude more security than the original method. Therefore, it can be concluded that the improved scheme is superior to the original encryption scheme and is comparable other similar schemes.

Of course, although the algorithm in this paper improves security, it also has some disadvantages; for example, it also slightly increases the processing time, and with the image size increases, the running time difference also expands. Our future research direction is to, on the basis of the chaos and security of this paper, improve the performance, so that the operation speed of encryption is faster and the time complexity is lower.

## Figures and Tables

**Table 1:** Correlation coefficients in different directions by different algorithms.

Image	Direction	Plain Image	Original	Improved
	Horizontal	0.9448	−0.0322	0.0139
Lena	Vertical	0.9711	−0.0221	0.0103
	Diagonal	0.9175	0.0139	−0.0183
	Horizontal	0.9192	0.0197	0.0115
Airfield	Vertical	0.9207	−0.0200	0.0165
	Diagonal	0.8731	0.0036	0.0262
	Horizontal	0.8629	0.0176	0.0246
Dollar	Vertical	0.7958	0.0016	−0.0073
	Diagonal	0.7222	−0.0262	0.0191
	Horizontal	0.9673	0.0132	−0.0133
Pepper	Vertical	0.9752	0.0060	−0.0089
	Diagonal	0.9443	−0.0160	0.0098

**Table 2:** Information entropies of different images based on different algorithms.

Image	Plain Image	Original	Standard Error	Improved	Standard Error
Lena	7.4116	7.9958	0.0042	7.9970	0.0030
Airfield	7.6407	7.9965	0.0035	7.9967	0.0033
Dollar	6.9983	7.9965	0.0035	7.9969	0.0031
Pepper	7.5808	7.9967	0.0033	7.9965	0.0035
Mean Value		7.9964	0.0036	7.9968	0.0032

**Table 3:** NPCR and UACI values of different images by different algorithms.

**Image**	**NPCR (%)**	
	**Original**	**Improved**
Lena	99.60	99.57
Airfield	99.66	99.57
Dollar	99.60	99.61
Pepper	99.59	99.56
	**UACI (%)**	
	**Original**	**Improved**
Lena	33.67	33.75
Airfield	33.87	33.61
Dollar	33.63	33.77
Pepper	33.61	33.98

**Table 4:** Execution time of original plan and improved plan.

Image	Execution Time (/s)	
	Original	Improved
Lena	16.8901	16.9165
Airfield	16.5361	16.9421
Dollar	18.4913	18.5421
Pepper	16.6588	16.6956
Mean Value	17.1441	17.2740

## Data Availability

All results and data obtained can be found in open access publications.
